# Survival from cancers of the kidney and ureter in England and Wales up to 2001

**DOI:** 10.1038/sj.bjc.6604601

**Published:** 2008-09-23

**Authors:** S Westlake, N Cooper, B Rachet, M P Coleman

**Affiliations:** 1 Social and Health Analysis and Reporting Division, Office for National Statistics (Room FG/114), 1 Myddelton Street, London EC1R 1UW, UK; 2 Cancer Research UK Cancer Survival Group, Non-Communicable Disease Epidemiology Unit, Department of Epidemiology and Population Health, London School of Hygiene and Tropical Medicine, Keppel Street, London WC1E 7HT, UK

Malignant neoplasms of the kidney and ureter comprise around 2% of adult cancers in England and Wales, some 5000 new cases a year. Cancers of the kidney account for some 90% of upper urinary tract malignancies. They are analysed together here, and will often be referred to as kidney cancer for brevity. Incidence is two to three times higher in Europe and North America than in Africa, Asia or Latin America (Ferlay *et al*, 2004).

Cancers of the kidney and ureter are twice as common in men. They are uncommon before the age of 50, with age-specific incidence rising steeply above that age, especially in men. Incidence rates increased by about 50% in England and Wales to 10 and 6 per 100 000 per year in men and women, respectively, between the mid-1980s and 1999 (Quinn *et al*, 2001). There was no systematic difference between socioeconomic groups in incidence or in incidence trends during the period 1986–1999 (data not shown). Mortality rates for kidney cancer in England and Wales have remained fairly stable over the last decade (Quinn *et al*, 2001).

Renal cell adenocarcinoma typically accounts for 80% or more of renal cancers, and urothelial (transitional cell) carcinoma of the renal pelvis and ureter accounts for most of the remainder (Harris and Simons, 2004). Wilms' tumour (nephroblastoma) is the most common renal tumour in children, but it is very rare in adults (less than 0.2 per million per year) (Mitry *et al*, 2006).

Obesity, smoking and hypertension are associated with an increased risk of renal cell carcinoma (Lipworth *et al*, 2006). Smoking is also a risk factor for urothelial carcinoma of the renal pelvis and ureter (McLaughlin *et al*, 1992), as is occupational exposure to petrochemicals.

The triad of flank pain, abdominal mass and haematuria has been the basis of clinical diagnosis, but incidental diagnosis during abdominal imaging has become more common, and it may underlie part of the increase in recorded incidence. Non-specific symptoms and the retroperitoneal location of the kidneys make early clinical diagnosis difficult. Less than half of patients have disease confined to the kidney at diagnosis, and a quarter present with metastatic disease, often in lung or bone. Nephrectomy is the main treatment for renal carcinoma, with interleukin-2 and interferon for advanced disease (Harris and Simons, 2004).

We analysed the survival of 49 721 adults (63% men) who were diagnosed with a first, primary, invasive neoplasm of the kidney or ureter in England and Wales during the 14-year period 1986–1999 and followed up to the end of 2001, about 81% of the 61189 patients eligible for analysis. Some 12% of patients were excluded from analysis because their recorded survival was zero (date of diagnosis same as the date of death): most of these will have been registered from a death certificate only (DCO), but in these data, they could not be reliably distinguished from patients with true zero survival. Other patients were excluded because the kidney cancer was not their first primary cancer (4.6%), or because their vital status was unknown on 5 November 2002, when the data were extracted for analysis (1.5%).

Renal cell carcinoma accounted for about 87% of kidney cancers analysed. Around 10% were transitional cell carcinomas of the renal pelvis or ureter, and the remainder were coded to other or unspecified urinary organs (data not shown).

## Survival trends

**Figure 1:**
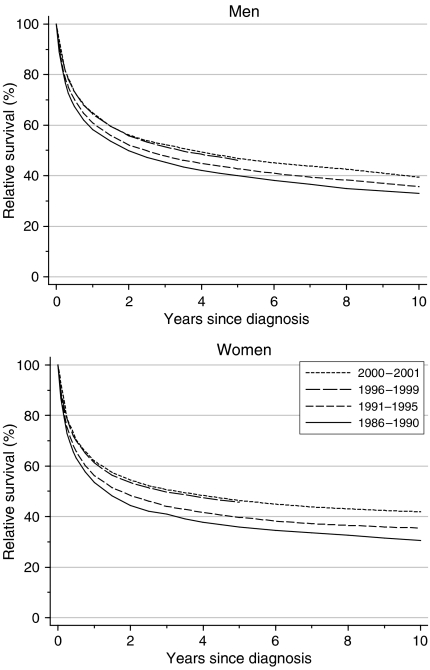
Relative survival (%) up to 10 years after diagnosis by sex and calendar period of diagnosis: England and Wales, adults (15–99 years) diagnosed during 1986–1999 and followed up to 2001. Survival estimated with cohort or complete approach (1986–1990, 1991–1995, 1996–1999) or hybrid approach (2000–2001) (see Rachet *et al*, 2008).

Relative survival from kidney cancer at 1 year was 58% for men and 54% for women diagnosed during 1986–1990, rising steadily to 65 and 61%, respectively, for those diagnosed during 1996–1999 (Table 1, Figure 1). For patients diagnosed during 1996–1999, 5-year survival had reached 46% in men and women. These changes represent a statistically significant deprivation-adjusted rate of increase of about 3% every 5 years for 1-year survival and 4–5% for 5-year survival. Improvements were slightly more marked in longer-term survival.

Short-term survival predictions, based on hybrid analysis (Brenner and Rachet, 2004) of the conditional probabilities of survival actually observed among men alive at some point during 2000–2001, suggest that survival up to 10 years after diagnosis will continue to improve, but somewhat more slowly, reaching 62–65% at 1 year, 46–47% at 5 years and 39–42% at 10 years, for patients diagnosed in 2000–2001 (Table 1, Figure 1).

## Deprivation

**Figure 2:**
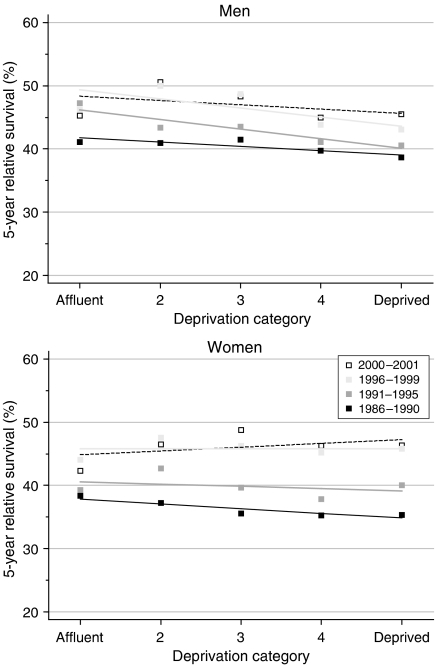
Trends in the deprivation gap in 5-year relative survival (%) by sex and calendar period of diagnosis: England and Wales, adults (15–99 years) diagnosed during 1986–1999 and followed up to 2001.

Among men, relative survival was systematically higher in more affluent groups, both at 1 year and at 5 years after diagnosis. The fitted difference in relative survival at 5 years between the most affluent and the most deprived groups (the deprivation gap) widened from −3% for those diagnosed in 1986–1990 to −6% for those diagnosed during 1996–1999, a statistically significant change of about −2% every 5 years (Table 2, Figure 2).

For women, in contrast, the deprivation gap in survival at both 1 and 5 years narrowed steadily during the 1990s, and was null or non-significant for those diagnosed during 1996–1999. Figure 2 shows the flattening of the gradient in 5-year survival.

Short-term predictions from hybrid analysis suggest that the deprivation gap in survival up to 5 years is not likely to increase further in the near future, and may fall slightly for men (Table 2).

## Comment

Survival from kidney cancer in England and Wales has increased steadily for both sexes during the last three decades. Relative survival at 5 years for patients diagnosed in 1971–1975 was only 30–31%, compared with around 46% for those diagnosed in the late 1990s. The pace of increase in survival has also improved, from around 3% every 5 years during the 1970s and 1980s (Coleman *et al*, 1999) to around 5% every 5 years in the 1990s, as seen in the data reported here.

Cancer of the kidney is unusual in that it is one of the few cancers for which men have tended to have a small survival advantage over women. Socioeconomic inequalities in survival are notably more marked among men, however, and they increased during the 1990s, whereas those for women have virtually disappeared. The explanation for these patterns is unclear.

Patients excluded from analysis as DCO cases may be biased with respect to survival (Berrino *et al*, 1995a), but the proportion of DCO cases excluded from analysis was the same in all categories of deprivation throughout the period 1986–1999 in both sexes (data not shown). The socioeconomic differences in survival, and the opposite trends in men and women, are thus unlikely to have been affected by these exclusions.

Five-year survival among patients diagnosed with kidney cancer in England up to the mid-1990s was among the lowest in Europe (Berrino *et al*, 1995b; Berrino *et al*, 1999; Berrino *et al*, 2003). Most of the improvement in survival in England and Wales occurred during the late 1990s, after the period of diagnosis covered by the EUROCARE-3 study (1990–1994).

## Figures and Tables

**Table 1:** Trends in relative survival (%) by sex, time since diagnosis and calendar period of diagnosis: England and Wales, adults (15–99 years) diagnosed during 1986–1999 and followed up to 2001

		**Calendar period of diagnosis^a^**						**Average change (%)**		**Prediction^c^ for patients**	
		**1986–1990**		**1991–1995**		**1996–1999**		**every 5 years^b^**		**diagnosed during 2000–2001**	
**Time since diagnosis**		**Survival (%)**	**95% CI**	**Survival (%)**	**95% CI**	**Survival (%)**	**95% CI**	**Survival (%)**	**95% CI**	**Survival (%)**	**95% CI**
1 year	Men	**58.3**	(57.2, 59.3)	**60.9**	(60.0, 61.9)	**64.5**	(63.5, 65.4)	2.6^**^	(0.7, 4.6)	**64.9**	(63.5, 66.2)
	Women	**53.5**	(52.1, 54.9)	**56.2**	(55.0, 57.4)	**61.2**	(60.0, 62.5)	3.0^*^	(0.4, 5.5)	**62.0**	(60.2, 63.7)
5 years	Men	**40.1**	(38.9, 41.2)	**42.8**	(41.7, 43.8)	**46.1**	(44.8, 47.4)	4.5^**^	(2.1, 6.8)	**46.9**	(45.3, 48.4)
	Women	**35.9**	(34.5, 37.4)	**39.7**	(38.4, 41.0)	**45.7**	(44.1, 47.3)	3.7^*^	(0.8, 6.7)	**46.3**	(44.3, 48.3)
10 years	Men	**33.0**	(31.8, 34.3)	**35.6**	(34.3, 37.0)			**4.4**	(−0.2, 9.0)	**39.4**	(37.6, 41.2)
	Women	**30.5**	(29.0, 32.0)	**35.4**	(33.9, 36.9)			5.9^*^	(0.4, 11.4)	**41.9**	(39.7, 44.1)

CI=confidence interval.

a Survival estimated with cohort or complete approach (see Rachet *et al*, 2008).

b Mean absolute change (%) in survival every 5 years, adjusted for deprivation (see Rachet *et al*, 2008).

c Survival estimated with hybrid approach (see Rachet *et al*, 2008).

^*^P<0.05; ^**^P<0.01.

**Table 2:** Trends in the deprivation gap in relative survival (%) by sex, time since diagnosis and calendar period of diagnosis: England and Wales, adults (15–99 years) diagnosed during 1986–1999 and followed up to 2001

		**Calendar period of diagnosis^a^**						**Average change (%)**		**Prediction^c^ for patients**	
		**1986–1990**		**1991–1995**		**1996–1999**		**every 5 years^b^**		**diagnosed during 2000–2001**	
**Time since diagnosis**		**Deprivation gap (%)**	**95% CI**	**Deprivation gap (%)**	**95% CI**	**Deprivation gap (%)**	**95% CI**	**Deprivation gap (%)**	**95% CI**	**Deprivation gap (%)**	**95% CI**
1 year	Men	−5.4^**^	(−8.5, −2.4)	−7.9^**^	(−10.5, −5.2)	−4.1^**^	(−6.9, −1.3)	**0.7**	(−1.5, 2.9)	**−2.7**	(−6.7, 1.3)
	Women	**−4.0**	(−7.9, 0.0)	**−0.6**	(−4.2, 2.9)	**−1.4**	(−5.0, 2.3)	**1.3**	(−1.5, 4.2)	**−1.9**	(−7.0, 3.3)
5 years	Men	**−2.7**	(−6.1, 0.6)	−6.0^**^	(−9.0, −3.1)	−5.8^**^	(−9.5, −2.0)	**−1.7**	(−4.3, 0.9)	**−2.7**	(−7.3, 1.8)
	Women	**−3.0**	(−7.1, 1.2)	**−1.4**	(−5.2, 2.3)	**0.0**	(−4.6, 4.6)	**1.6**	(−1.6, 4.8)	**2.4**	(−3.3, 8.1)
10 years	Men	**−1.0**	(−4.5, 2.6)	**−3.4**	(−7.2, 0.4)			**−2.4**	(−7.6, 2.8)	**−1.6**	(−6.7, 3.5)
	Women	**−1.3**	(−5.6, 3.0)	**−2.7**	(−7.1, 1.7)			**−1.4**	(−7.5, 4.7)	**0.5**	(−5.8, 6.8)

CI=confidence interval.

a Survival estimated with cohort or complete approach (see Rachet *et al*, 2008).

b Mean absolute change (%) in the deprivation gap in survival every 5 years, adjusted for the underlying trend in survival (see Rachet *et al*, 2008).

c Survival estimated with hybrid approach (see Rachet *et al*, 2008).

^**^P<0.01.
